# The influence of ezetimibe on classical and alternative activation pathways of monocytes/macrophages isolated from patients with hypercholesterolemia

**DOI:** 10.1007/s00210-014-0982-4

**Published:** 2014-04-30

**Authors:** Dariusz Suchy, Krzysztof Łabuzek, Grzegorz Machnik, Bogusław Okopień

**Affiliations:** Department of Internal Medicine and Clinical Pharmacology, Medical University of Silesia in Katowice, Medyków 18, 40752 Katowice, Poland

**Keywords:** Ezetimibe, Lipopolysaccharide, Monocyte, Macrophage activation, Hypercholesterolemia

## Abstract

Macrophages are crucial for the development of atherosclerotic plaques. Classically activated macrophages contribute to plaque growth and destabilization, while alternatively activated macrophages increase plaque stability. Here, we assessed the influence of ezetimibe on the activation of monocyte-derived macrophages isolated from patients with hypercholesterolemia (total cholesterol 263.4 ± 12.5 mg/dl, low-density lipoprotein cholesterol 179.7 ± 11.3 mg/dl, triglycerides 123.9 ± 11.4 mg/dl). Cells were stimulated with 1 μg/ml lipopolysaccharide (LPS) or 1 μg/ml LPS plus 22 ng/ml ezetimibe. Control cells were left unstimulated. The expression of classical activation markers (interleukin-1β (IL-1β), nitric oxide (NO), and inducible nitric oxide synthase (iNOS)) and alternative activation markers (mannose receptor (MR) and arginase-1 (Arg1)) was determined after 48 h. The employed analytical methods included enzyme-linked immunosorbent assay, Griess reaction, real-time polymerase chain reaction, and Western blotting. LPS increased the secretion of IL-1β and NO and the expression of iNOS mRNA, iNOS protein, and Arg1 protein. It did not affect the expression of MR or Arg1 mRNA. In comparison to LPS stimulation, co-stimulation with ezetimibe decreased the secretion of IL-1β and the expression of iNOS mRNA and protein, while it increased MR mRNA and protein expression. Co-stimulation with ezetimibe did not change the secretion of NO or the expression of Arg1. The results suggest that ezetimibe in inflammatory in vitro conditions contributes to the suppression of classical and promotion of the alternative macrophage activation.

## Introduction

Monocytes, along with granulocytes and natural killer cells, belong to the cellular elements of the innate, nonspecific immune system. Monocytes also link innate and adaptive immunity through antigen presentation and cytokine production (Auffray et al. 2009). After infiltration of surrounding tissues, monocytes differentiate into macrophages whose primary function is to uphold homeostasis by scavenging senescent cells and repairing injured areas undergoing inflammatory processes (Gordon and Taylor 2005). However, macrophages do not constitute a homogenous population. Depending on the stimulus, these cells undergo different activation and consequently present distinct properties. According to the most recent divisions, there are two major types of activated macrophages: the classically activated macrophages (M1) and the alternatively activated macrophages (M2). In vitro, there are in total five subpopulations: M1a, M1b, M2a, M2b, and M2c. However, there is no certainty regarding whether they constitute separate entities or if they represent subsequent stages of the development continuum. The M1a and M2a macrophages have also been identified in vivo. The existence of the other subpopulations in vivo is presently of minor probability (Rees 2010).

Macrophages are key cells involved in the process of atherogenesis (Auffray et al. 2009). As well as the differences in the roles of the given subpopulations, they also exert contrasting effects regarding the development and stability of the atherosclerotic plaque (Auffray et al. 2009; Wilson 2010). The M1 subpopulation effectively combats pathogens. However, the M1 macrophages also promote atherosclerotic plaque growth due to pro-inflammatory properties, damage of tissues, and absorption of low-density lipoproteins (LDLs). The M1 cells contribute to increased plaque vulnerability and susceptibility to cracking, which raises the risk of thrombotic changes, and hence this subpopulation is often called “atherogenic.” In contrast to the M1 subpopulation, the M2 macrophages produce anti-inflammatory cytokines, inhibit inflammatory reactions, and extinguish inflammatory states. The M2 macrophages promote tissue repair processes and stabilize atherosclerotic plaques, even leading to their regression, and hence the M2 macrophages are often referred to as being “atheroprotective” (Wilson 2010). Both M1 and M2 macrophages produce characteristic markers that are related to their functions. Among other markers, the M1 macrophages produce significant amounts of nitric oxide (NO) due to elevated expression of inducible nitric oxide synthase (iNOS), which is necessary for effective antimicrobial activity. They also secrete large quantities of interleukin-1β (IL-1β), a major pro-inflammatory cytokine (Gordon and Taylor 2005). On the other hand, the M2 macrophage subpopulation is characterized by increased activity of arginase-1 (Arg1), which is crucial during the final stages of inflammatory-state quenching. These cells also demonstrate a higher expression of mannose receptors (MRs), resulting in changes in phagocytosis and membrane transport (Varin and Gordon 2009).

Considering the properties of the macrophage subpopulations, investigations of the immunomodulative properties of various pharmacologically active substances are underway. From this point of view, the drugs used to treat hypercholesterolemia are of special interest. One of the newest therapeutic agents used to treat this disease is ezetimibe, a potent cholesterol and phytosterol absorption inhibitor that acts in the small intestine lumen. Ezetimibe blocks the Nieman-Pick C1-Like 1 (NPC1L1) transporter protein in the brush border of enterocytes (Suchy et al. 2011). At the daily oral dose of 10 mg, it reduces the absorption of cholesterol by about 54 % and decreases the plasma level of LDL cholesterol by about 15–20 %. Beside the influence on lipid parameters, ezetimibe also exerts additional effects such as reducing the level of C-reactive protein (CRP), which is a global inflammatory marker (Suchy et al. 2011). Ezetimibe also directly affects properties of monocytes and macrophages. The drug modifies the functioning of monocyte lipid rafts and reduces the absorption of oxidized LDLs in macrophages. In this way, it reduces the formation of foam cells and the gathering of lipids in the atherosclerotic plaques. Ezetimibe in macrophages undergoes endocytosis and, after being absorbed into these cells, is also able to modify the expression of several genes (Suchy et al. 2011). However, whether the drug influences macrophage activation pathways has not yet been investigated.

The aim of this study was to assess the in vitro impact of ezetimibe on the activation of monocyte-derived macrophages isolated from patients with hypercholesterolemia and thereby its potential stabilizing effect on the atherosclerotic plaque.

## Methods

### Materials

Ezetimibe, (3*R*,4*S*)-1-(4-fluorophenyl)-3-[(3*S*)-3-(4-fluorophenyl)-3-hydroxypropyl]-4-(4-hydroxyphenyl)-azetidin-2-one, was purchased from LKT Laboratories, Inc. (USA). Lipopolysaccharide (LPS, *Escherichia coli* serotype 0111:B4), polysaccharose Histopaque 1077, dimethyl sulfoxide (DMSO) Hybri-Max, trypan blue 0.4 % solution, TRIzol/TRI reagent, chloroform, isopropanol, protease inhibitor cocktail, Bradford reagent, Laemmli sample buffer concentrate, 40 % bis-acrylamide, tetramethylethylenediamine (TEMED), Trizma base (Tris), sodium dodecyl sulfate (SDS), ammonium persulfate (APS), sodium chloride, hydrochloric acid, Tween 20, glycine, bovine serum albumin (BSA), and primary rabbit anti-human antibody anti-Arg1 (307-322) were from Sigma-Aldrich Co. (USA). RPMI 1640 (without phenol red, with glutamine), 1 M 4-(2-hydroxyethyl)-1-piperazineethanesulfonic acid (HEPES) buffer, Hank's balanced salt solution (HBSS; without calcium, magnesium, and phenol red), and Dulbecco’s phosphate-buffered saline (PBS) were bought from PAA Laboratories GmbH (Austria). Glutamine was purchased from Gibco-BRL (USA). Low-endotoxin fetal bovine serum (FBS), antibiotic-antimycotic anti-anti solution, and glycogen were from Invitrogen (USA). Absolute ethanol and methanol were bought from Merck Millipore (Germany). Magnetic beads (pan-T (anti-CD2) and pan-B (anti-CD19)) were purchased from Dynal Biotech ASA (Norway). Monoclonal mouse anti-human antibody anti-CD14 (CD14-FITC) was bought from Thermo Fisher Scientific Inc. (USA). Nitrate/Nitrite Colorimetric Assay Kit was purchased from Cayman Chemical Company (USA). The enzyme-linked immunosorbent assay (ELISA) kit for human IL-1β was from R&D Systems, Inc. (USA). Primers (Table 1) for the real-time polymerase chain reaction (RT-PCR) were obtained from Genomed S.A. (Poland). Affinity Script QPCR cDNA Synthesis Kit and PCR Brilliant II SYBR Green QPCR Master Mix with Low ROX were purchased from Agilent Technologies (USA). ColorPlus Prestained Protein Markers (Broad Range (97–175 kDa) P7709V and Broad Range (10–230 kDa) P7711S) were from New England Biolabs Inc. (USA). Radioimmunoprecipitation assay (RIPA) lysis buffer, SuperSignal Molecular Weight Protein Ladder, Western blot polyvinylidene fluoride (PVDF) transfer membrane, primary rabbit anti-human antibody anti-β-actin (PA1-21167), and Restore Plus Western Blot stripping buffer were from Thermo Fisher Scientific Inc. (USA). Primary rabbit anti-human antibodies anti-MR (ab64693) and anti-iNOS (ab15323) were from Abcam (USA). Amersham ECL Western Blotting Analysis System was purchased from GE Healthcare (UK).

**Table 1:** Sequences of primers used in the RT-PCR

Gene	Sequences of primers
iNOS	Forward: CCATGTCTGGGAGCATCAC
	Reverse: GCATACAGGCAAAGAGCACA
MR	Forward: TTTTTCCTTTGCCTAATTGAAGA
	Reverse: GCTGACATCAGCTACCCATC
Arg1	Forward: GACCCTGGGGAACACTACATT
	Reverse: GTGCCAGTAGCTGGTGTGAA
GAPDH	Forward: GAAGGTGAAGGTCGGAGTC
	Reverse: GAAGATGGTGATGGGATTTC

*Arg1* arginase-1, *GAPDH* glyceraldehyde 3-phosphate dehydrogenase, *iNOS* inducible nitric oxide synthase, *MR* mannose receptor

### Isolation of human monocytes

The study was accepted by the Bioethical Committee of the Medical University of Silesia in Katowice, Poland. The investigation conformed to the principles of the Declaration of Helsinki. Human whole-blood samples were taken from 20 yet untreated patients with primary hypercholesterolemia (total cholesterol 263.4 ± 12.5 mg/dl, LDL cholesterol 179.7 ± 11.3 mg/dl, triglycerides 123.9 ± 11.4 mg/dl) registered at the Independent Public Central Clinical Hospital of the Medical University of Silesia in Katowice, Poland (Table 2). Later therapy of the patients was not altered as the samples were taken only once. Peripheral blood monocytic cells (PBMCs) were obtained by density-gradient centrifugation using polysaccharose (Okopień et al. 2005). Monocytes were isolated from the mixed population of PBMCs using the negative immunomagnetic method (Flø et al. 1991), which excludes T, B, and NK cells with magnetic beads coated with the relevant antibodies. The mean (±standard deviation, SD) purity of the obtained monocytes determined by fluorescent antibody labeling with anti-CD14 was 92.02 ± 2.37 %. Next, the monocytes obtained from each of the patients were diluted in HBSS containing 2 % (*v*/*v*) FBS to obtain a cell density of 1 × 10^6^/ml. Three 1-ml suspension portions were placed in a 24-well culture dish (Nunclon Delta Surface, Nunc, Denmark) and incubated for 3 h at 37 °C in air containing 5.2 % (*v*/*v*) carbon dioxide and 95 % (*v*/*v*) relative humidity in order to allow the monocytes to adhere. After that time, the cells were gently washed to remove the remaining nonadherent cells. Three wells containing cells from one patient constituted one series of cultures. The cells in each of the wells underwent different stimulations.

**Table 2:** Characteristics of participants

Parameter	Value
Sex (females/males)	9/11
Age ± SD (years)	53.6 ± 3.9
Body mass index (kg/m^2^)	28.2 ± 1.9
Cigarette smokers (%)	30.0
Total cholesterol (mg/dl)	263.4 ± 12.5
LDL cholesterol (mg/dl)	179.7 ± 11.3
HDL cholesterol (mg/dl)	43.1 ± 2.5
Triglycerides (mg/dl)	123.9 ± 11.4

*HDL* high-density lipoprotein, *LDL* low-density lipoprotein, *SD* standard deviation

### Cell cultures

Monocytes were cultured in RPMI 1640 supplemented with 10 % (*v*/*v*) FBS, 25 mM HEPES, 2 mM glutamine, 100 U/ml penicillin, 100 μg/ml streptomycin, and 10 μg/ml amphotericin B as in the above conditions. The cells were adapted to the conditions for 24 h, after which the medium was replaced with either (1) regular medium, (2) medium supplemented with 1 μg/ml (submaximal dose) LPS, or (3) medium supplemented with 1 μg/ml LPS plus 22 ng/ml ezetimibe. The concentration of ezetimibe was equivalent to that measured in the serum of patients chronically taking the drug at the oral daily dose of 10 mg (Suchy et al. 2013). Ezetimibe as a lipophilic substance was dissolved in DMSO to facilitate its introduction into the aqueous culture medium (Cheng et al. 2009). The final concentration of DMSO in the culture media (including those not supplemented with ezetimibe solution) was equal to 0.05 % (*v*/*v*). The cells were incubated for 48 h, after which cell culture supernates were collected and the cells were harvested by vigorous pipetting with ice-cold PBS. The cell culture supernates were centrifuged and immediately frozen at −80 °C. They were subsequently used for the determination of NO and IL-1β concentrations. The harvested cells from each well were divided into two portions: one was subjected to Western blotting analysis and the other to RT-PCR analysis in order to determine the expression of iNOS, Arg1, and MR.

### Assessment of cell viability

The viability of the cells was examined after the isolation of monocytes from human whole-blood samples (to evaluate their suitability for culture) and after harvesting the macrophages (to determine whether the results were affected by disturbances during incubation). The viability was assessed by staining the cells with trypan blue solution. The suspended cells were incubated for 5 min in the above conditions with 0.2 % (*v*/*v*) dye diluted in the cell culture medium. After that time, the percentage of dead, blue-stained cells was counted using a Bürker's chamber.

### Assessment of NO production

The concentration of NO produced by the cells after different stimulations was calculated as the sum of nitrites and nitrates, which are its stable derivatives. The assessments were carried out according to the instructions of the kit manufacturer. Briefly, initially all the cell culture supernates were diluted twofold. Next, the nitrates present in 80-μl samples were reduced into nitrites with the use of nitrate reductase. The amount of nitrites in the samples of culture media was determined through reaction with an equal volume of Griess reagent. After 10 min of incubation, the absorbances at 540 nm were measured in a microplate reader (xMark Microplate Spectrophotometer, Bio-Rad Laboratories, Inc., USA). Fresh culture medium was used as a blank in all the measurements. The amount of nitrites in the samples was calculated using the calibration curve based on serial dilutions of sodium nitrite standard.

### Assessment of IL-1β secretion

The concentration of IL-1β produced by the cells after different stimulations was measured using a commercially available ELISA kit according to the manufacturer's instructions. Briefly, 200-μl samples of cell culture supernates were incubated in the anti-IL-1β-antibody-covered wells of a 96-well plate. After washing the wells, equal volumes of antibody conjugate were added. Subsequently, the wells were washed again and substrate solutions were added. After stopping the reaction, the absorbances at 450 nm (with correction at 540 nm) were measured in a microplate reader (xMark Microplate Spectrophotometer, Bio-Rad Laboratories, Inc., USA). The amount of IL-1β in the samples was calculated using the calibration curve based on serial dilutions of IL-1β standard.

### Assessment of iNOS, Arg1, and MR mRNA expression

The expression of marker genes was analyzed using the RT-PCR method. Total RNA was extracted with TRIzol reagent according to the manufacturer's instructions based on a previously developed method (Chomczyński and Sacchi 1987). The RNA extracts were supplemented with 2 μg of glycogen. Reverse transcription was performed in a MJ Mini Personal Thermal Cycler (Bio-Rad Laboratories, Inc., USA) with 0.5 μg total RNA using a cDNA synthesis kit according to the manufacturer's instructions. RT-PCR was performed using SYBR Green QPCR Master Mix with Low ROX with a RT-PCR LightCycler 480 II (Roche Applied Science, USA). PCR primers for iNOS (E.C. 1.14.13.39), Arg1 (E.C. 3.5.3.1), and MR used in this study are listed in Table 1. Glyceraldehyde 3-phosphate dehydrogenase (GAPDH, E.C. 1.2.1.12) was used as a reference gene. The relative expression values were calculated using the comparative threshold cycle (ΔΔ*C*_t_) method.

### Assessment of iNOS, Arg1, and MR protein expression

The expression of marker proteins was analyzed using the Western blotting method. Whole-cell extracts were obtained by lysing the cells with RIPA buffer with the addition of protease inhibitors (104 mM AEBSF, 80 μM aprotinin, 4 mM bestatin, 1.4 mM E-64, 2 mM leupeptin, 1.5 mM pepstatin A in the stock solution). The concentration of proteins was assessed using Bradford's reagent (Bradford 1976) by reading the absorbance at 595 nm in a microplate reader (xMark Microplate Spectrophotometer, Bio-Rad Laboratories, Inc., USA) and comparing the values with a calibration curve based on serial dilutions of BSA. Equal amounts (25 μg) of total proteins were run on SDS-PAGE (10 % separating gel) in a Mighty Small II electrophoretic chamber (Hoefer, USA) and transferred to a PVDF membrane in a TE22 Mighty Small Transphor Unit (Hoefer, USA). The membrane was blocked for 1 h at room temperature in 1 % (*v*/*v*) BSA solution in Tris-buffered saline containing 0.08 % (*v*/*v*) Tween 20 (TTBS). After blocking, the membrane was incubated with subsequent specific primary antibodies (dilution 1:1,000) for 1 h at room temperature and further incubated for 1 h with a secondary antibody (dilution 1:10,000) conjugated with horseradish peroxidase. The addition of substrate solution resulted in the formation of a chemiluminescent product. The chemiluminescence was read in a ChemiDoc-It 410 camera (UVP, USA) and analyzed densitometrically. β-Actin was used as a reference protein. Independent expression analyses of respective proteins (iNOS, Arg1, MR, and β-actin) were preceded by membrane stripping to remove the adsorbed primary antibodies.

### Statistical analysis

The statistical analysis was performed with GraphPad Prism 6 for Windows (GraphPad Software Inc., USA). The normality of continuous variable distributions was checked with the Shapiro-Wilk's test. The results are presented as means ± SD. Significant differences between experimental groups were determined using the one-way ANOVA analysis with Tukey's post hoc test. The homogeneity of variance, which is an assumption of ANOVA, was checked with Brown-Forsythe’s and Bartlett’s tests. The differences were considered significant at the level of *p* < 0.05. The levels of significance are marked as follows: *p < 0.05, **p < 0.01, ***p < 0.001, and ****p < 0.0001. The sample sizes (number of replicates, *n*) are given with the relevant analysis results. The *n* values lower than 20 (approved by the Bioethical Committee) resulted from independent external causes affecting the performance of the experiment, resulting in the rejection of the whole series.

## Results

### The influence of culture preparation procedures and different types of stimulation on the viability of the cells

The viability of isolated monocytes was assessed each time before beginning a series of cultures. The mean viability of the cells obtained from all the participants of the experiment (*n* = 20) was 96.42 ± 3.35 %.

The viability of cultured cells, including the control cultures, the cultures stimulated with LPS (1 μg/ml), and stimulated with LPS (1 μg/ml) plus ezetimibe (22 ng/ml) (*n* = 3 for each), was tested at the beginning, during, and at the end of the study. The mean viabilities of macrophages from the respective types of cultures were 95.08 ± 5.09, 93.16 ± 4.51, and 94.86 ± 4.04. The given values were not significantly different (*p* = 0.9784), indicating that neither LPS nor ezetimibe at the concentrations used affected the cell viability. The results of viability testing also indicated that this parameter did not have any influence on the outcome of the experiments.

### LPS promotes the classical activation pathway of monocyte-derived macrophages isolated from hypercholesterolemic patients

**Fig. 1:**
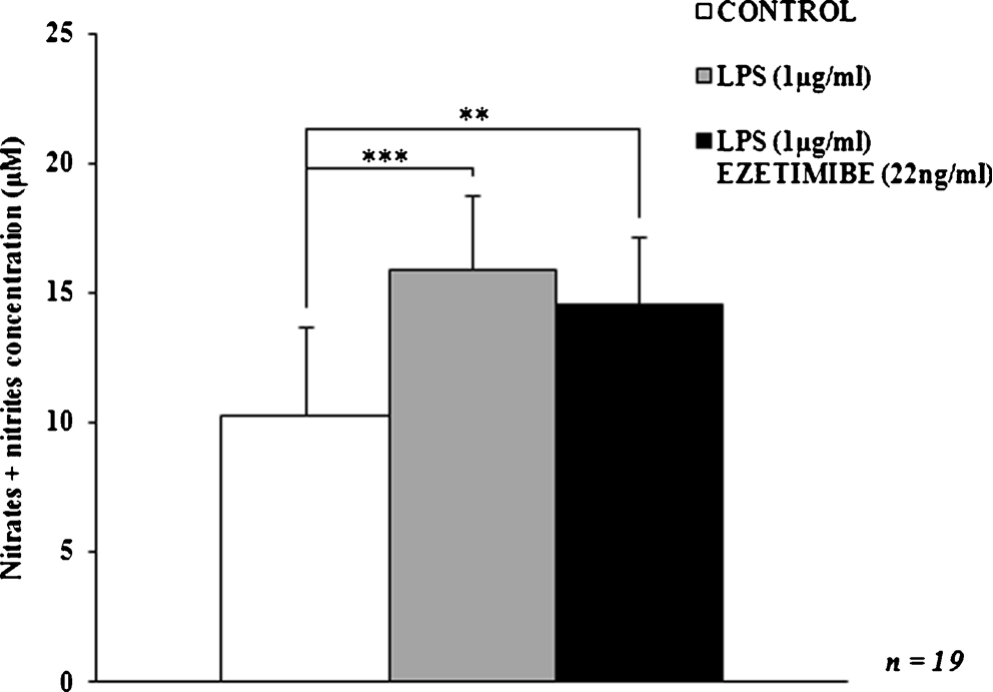
The influence of ezetimibe and/or LPS on the concentration of NO derivatives. The cells were treated with LPS (1 μg/ml) or LPS (1 μg/ml) plus ezetimibe (22 ng/ml) or were left untreated (control), for 48 h. The total concentration of nitrates and nitrites produced by the cells was measured by the Griess reaction. Each value represents mean ± SD. The sample size (*n*) represents the number of replicates measured for each of the markers in each group in the given experiment. The levels of significance are marked as follows: *p < 0.05; **p < 0.01; ***p < 0.001; ****p < 0.0001

**Fig. 2:**
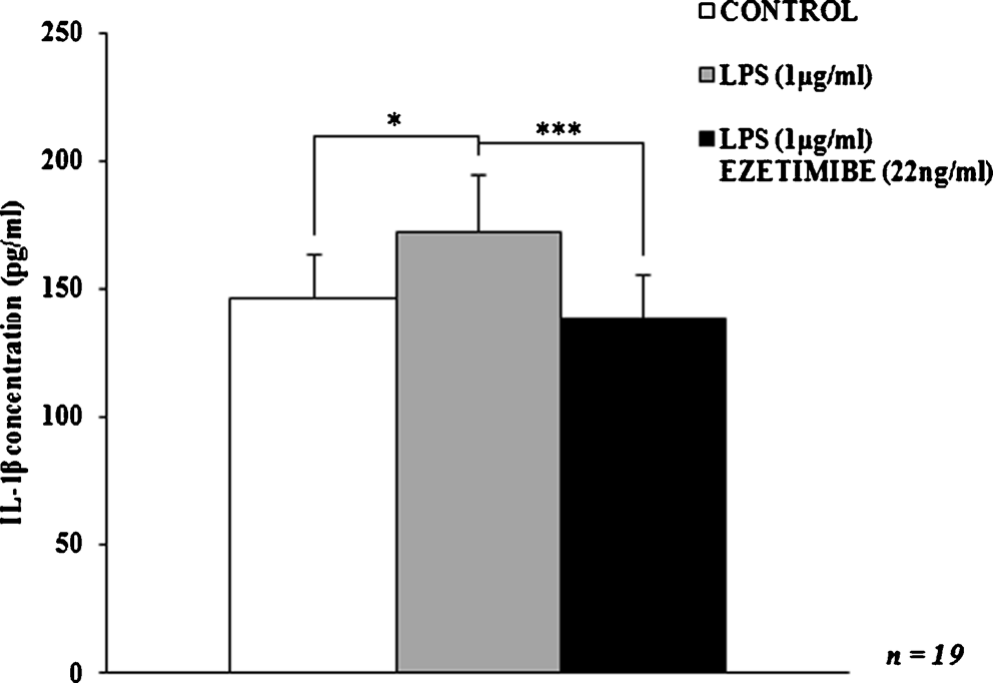
The influence of ezetimibe and/or LPS on the concentration of IL-1β. The cells were treated with LPS (1 μg/ml) or LPS (1 μg/ml) plus ezetimibe (22 ng/ml) or were left untreated (control), for 48 h. The concentration of secreted IL-1β was assessed by ELISA. Each value represents mean ± SD. The sample size (*n*) represents the number of replicates measured for each of the markers in each group. The levels of significance are marked as follows: *p < 0.05; **p < 0.01; ***p < 0.001; ****p < 0.0001. *IL*-*1β* interleukin-1β

**Fig. 3:**
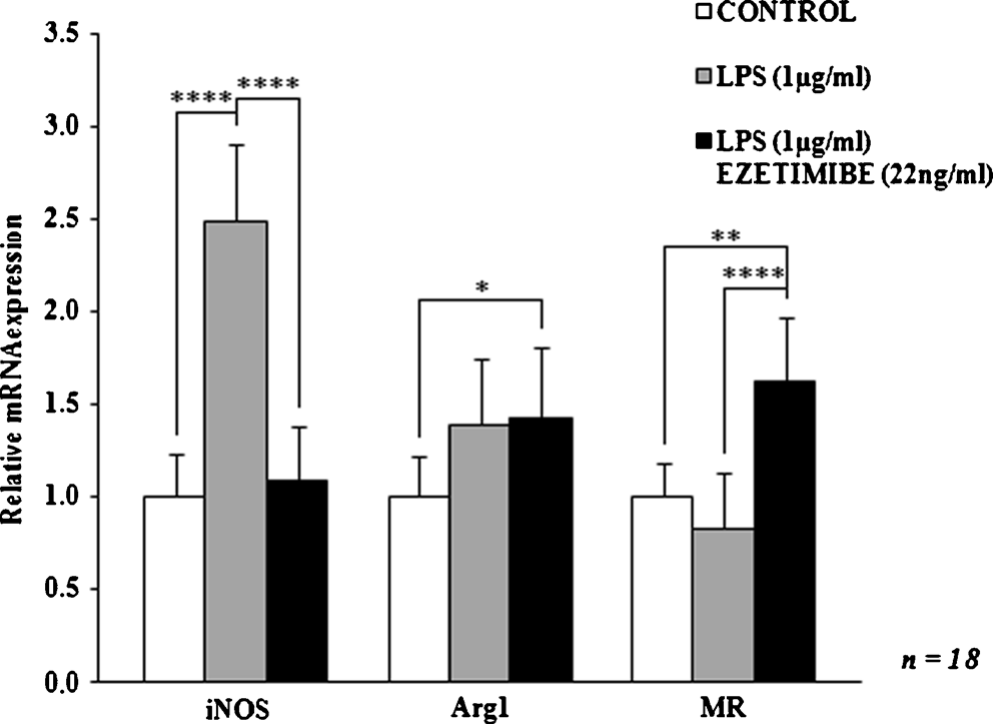
The influence of ezetimibe and/or LPS on the mRNA expression of macrophage activation markers. The cells were treated with LPS (1 μg/ml) or LPS (1 μg/ml) and ezetimibe (22 ng/ml) or were left untreated (control), for 48 h. The mRNA levels of iNOS, Arg1, and MR were determined by RT-PCR and analyzed by the comparative threshold cycle (ΔΔ*C*_t_) method. GAPDH was used as a housekeeping gene. Each value represents mean ± SD. The sample size (*n*) represents the number of replicates measured for each of the markers in each group in the given experiment. The levels of significance are marked as follows: *p < 0.05; **p < 0.01; ***p < 0.001; ****p < 0.0001. *GAPDH* glyceraldehyde 3-phosphate dehydrogenase, *iNOS* inducible nitric oxide synthase, *Arg1* arginase-1, *MR* is mannose receptor

**Fig. 4:**
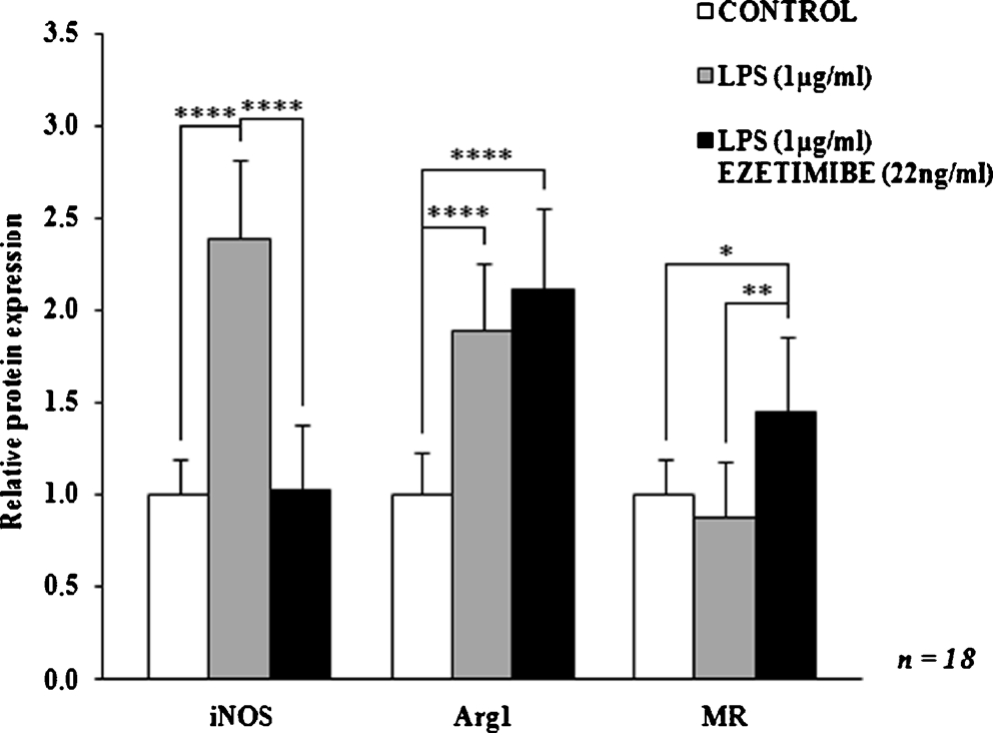
The influence of ezetimibe and/or LPS on the protein expression of macrophage activation markers. The cells were treated with LPS (1 μg/ml) or LPS (1 μg/ml) plus ezetimibe (22 ng/ml) or were left untreated (control), for 48 h. The protein levels of iNOS, Arg1, and MR were determined by Western blotting and analyzed densitometrically. β-Actin was used as a reference protein. Each value represents mean ± SD. The sample size (*n*) represents the number of replicates measured for each of the markers in each group. The levels of significance are marked as follows: *p < 0.05; **p < 0.01; ***p < 0.001; ****p < 0.0001. *iNOS* inducible nitric oxide synthase, *Arg1* arginase-1, *MR* mannose receptor

**Fig. 5:**
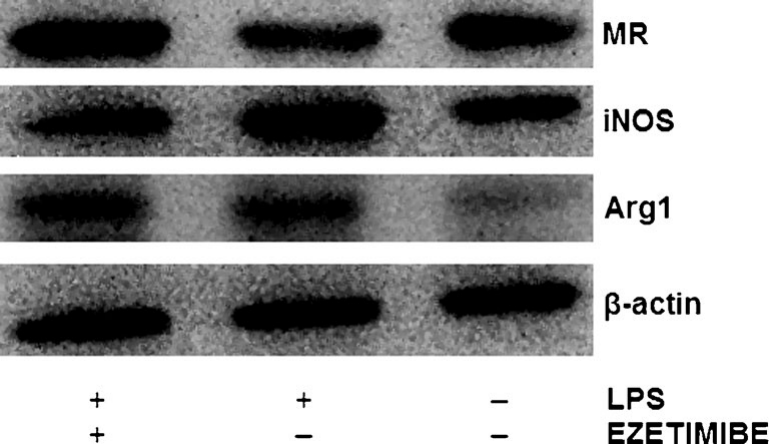
Representative Western blot image. *Arg1* arginase-1, *iNOS* inducible nitric oxide synthase, *LPS* lipopolysaccharide, *MR* mannose receptor

The stimulation of macrophages with LPS significantly increased expression of classical activation markers. LPS treatment raised the total amount of nitrates and nitrites in the cell culture supernates by 55 % (Fig. 1). Additionally, we observed an 18 % elevation in IL-1β concentration (Fig. 2). The increase in NO production was accompanied by the upregulation of its main producer, iNOS. The expression of the enzyme was increased by more than twofold, both at the mRNA and protein levels. In contrast, the levels of alternative macrophage activation markers under the influence of LPS remained unaffected with the exception of the expression of Arg1 protein, which was significantly elevated. Concomitantly, the expression of Arg1 mRNA was higher in comparison to the control cells but the difference was not significant. The expression of MR was not altered significantly. Both mRNA and protein levels of MR were about 15 % lower (Figs. 3, 4, and 5). These observations suggest that LPS promotes the classical activation of macrophages obtained from hypercholesterolemic patients, which may resemble the state occurring in growing atherosclerotic plaques.

### Ezetimibe silences the classical activation pathway of monocyte-derived macrophages isolated from hypercholesterolemic patients

Co-stimulation of cultured macrophages with LPS and ezetimibe decreased the expression of classical activation markers. The most significant change regarded the expression of iNOS mRNA and protein: the relative expression values were decreased by over 50 % in comparison to the expression observed in LPS-stimulated cells. The expression of iNOS mRNA and protein was comparable to that of control macrophages (*p* = 0.8260 and *p* = 0.9862, respectively; Figs. 3, 4, and 5). The reduction of iNOS expression was accompanied by changes in NO secretion. The concentration of NO derivatives in the co-stimulated macrophages was 9 % lower than in the LPS-stimulated cells (Fig. 1). However, the observed difference was not significant (*p* = 0.5844). The amount of generated NO was significantly higher (43 %) than in the control group, but the difference was less significant (*p* < 0.01 vs. *p* < 0.001). Simultaneous stimulation of macrophages with LPS and ezetimibe resulted in a 20 % reduction in the secretion of IL-1β compared to the LPS-stimulated cells (Fig. 2). The amount of the cytokine measured in the supernates from the co-stimulated cells was comparable to that of the control cells (6 % lower, *p* = 0.6117). The above results indicate that the addition of ezetimibe to the culture medium silences the classical pathway of macrophage activation evoked by LPS.

### Ezetimibe contributes to the alternative activation pathway of monocyte-derived macrophages isolated from hypercholesterolemic patients

The co-stimulation of macrophages with LPS and ezetimibe increased the expression of alternative activation markers. The addition of LPS to the medium resulted in the elevation of Arg1 mRNA expression; however, it was the addition of ezetimibe that induced a statistically significant change. The expression of Arg1 mRNA in the co-stimulated cells was 42 % higher than in the control group. Also, the expression of Arg1 protein was significantly increased (more than twofold) compared to the control cells. Of note, the expression of Arg1 was only slightly higher than in the LPS-stimulated macrophages. The most evident changes were observed regarding the MR. The expression of MR in the co-stimulated cells compared to the control cells was 63 and 45 % higher at the mRNA and protein levels, respectively. The elevation was even more pronounced when compared to the LPS-stimulated cells: the expression of the MR mRNA was almost twofold higher and the expression of the MR protein was 66 % higher (Figs. 3, 4, and 5). The noticed alterations suggest that ezetimibe at the concentration that can be found in serum after chronic administration promotes the alternative activation of macrophages obtained from hypercholesterolemic patients.

## Discussion

Macrophages gathering in the arterial walls are key cells in the pathogenesis of atherosclerotic lesions. Depending on their activation type, macrophages may have opposing impacts on the development and stability of plaques (Łabuzek et al. 2013; McLaren et al. 2011). Atherosclerosis is a major complication in hypercholesterolemia, and thus we decided to isolate monocytes from newly diagnosed hypercholesterolemic patients. During several days of incubation (Lovren et al. 2010; Zhou and Amar 2007) and LPS stimulation (Lovren et al. 2010; Panda et al. 2012), the monocytes were differentiated into macrophages. The use of LPS at a submaximal concentration of 1 μg/ml was intended to imitate the inflammatory state of atherosclerotic lesions, to induce M1 polarization, and to facilitate the determination of the qualitative and quantitative effects of ezetimibe. In this way, the effect of priming the macrophages with the chosen LPS concentration could potentially be decreased or increased by the study drug. We decided not to use common macrophage-differentiating factors such as granulocyte-macrophage colony-stimulating factor (GM-CSF) or macrophage colony-stimulating factor (M-CSF) as they have been reported to induce M1 and M2 polarization, respectively, but do not have a direct influence on inflammatory-state generation (Fleetwood et al. 2009; Van der Plas et al. 2009). We also did not use phorbol-12-myristate-13-acetate (PMA) based on information that ezetimibe impairs the differentiation process evoked by PMA (Muñoz-Pacheco et al. 2012). Furthermore, we did not evaluate the effect of ezetimibe alone as atherosclerosis is an inflammatory disease and—being aware of the model's imperfections—we chose the co-stimulation to resemble the actual conditions in which the drug is therapeutically used.

The markers used to determine the phenotype of macrophages were chosen based on their significance for the process of atherogenesis and/or their credibility. IL-1β is not only a well-accepted M1 macrophage marker but also contributes to M1 macrophage generation. The cytokine also promotes foam-cell formation because it reduces ABCA1/ABCG1 transporter mRNA expression and increases intracellular lipid deposition. It was found to potentiate atherosclerotic changes in mice in vivo (McLaren et al. 2011). iNOS is present in tissues undergoing inflammatory processes, including atherosclerotic lesions, and its expression magnitude correlates with severity of the disease. The in vitro experiments revealed that NO produced by iNOS in activated macrophages causes apoptotic and necrotic cell death (Tripathi 2007). Peroxynitrites originating from NO induce protein nitration and apoptosis of vascular smooth muscle cells (VSMCs), which in turn leads to atherosclerotic plaque instability. Increased NO production in M1 macrophages worsens atherogenic conditions. On the other hand, iNOS expression blockade in hypercholesterolemic mice suppresses atherogenesis development (Feig and Feig 2012). In turn, Arg1 is responsible for promitotic properties of M2 macrophages, i.e., inducing VSMC proliferation, leading to increased atherosclerotic plaque stability. Arg1 competes with iNOS for the common substrate l-arginine (l-Arg). Arg1 metabolizes l-Arg into l-ornithine, which is a precursor of polyamines and proline. Polyamines play a crucial role in cell growth, differentiation, and division, while proline is a key component of collagen. Arg1 expression is typical for the M2 macrophages (Khallou-Laschet et al. 2010). Arg1 is currently regarded as a new therapeutic target in atherosclerosis prevention. In the experiments on animals, it reduced atherosclerotic processes irrespective of the LDL level. In contrast, generation of foam cells was linked with suppression of Arg1 expression (Feig and Feig 2012). MRs are reliable markers of alternative macrophage activation in humans and mice (Martinez-Pomares 2012). It was observed that macrophages expressing MR were localized in regions far from the lipidous necrotic core of atherosclerotic plaque but their presence in the core itself was significantly lower. During in vitro investigations, these macrophages were filled with lipid droplets smaller than macrophages not expressing MR, which demonstrated their decreased ability to scavenge LDL and oxidized LDL and, in turn, their lower probability of developing into foam cells (Chinetti-Gbaguidi et al. 2011).

By modulating macrophage activation, the dynamics of atherosclerosis progression can be altered (Johnson and Newby 2009; Khallou-Laschet et al. 2010). Previous investigations proved that inhibition of the classical activation pathway with CD40 antagonists and induction of the alternative activation with peroxisome proliferator-activated receptor-γ (PPARγ) agonists contributes to a restriction of atherosclerotic processes (Lutgens et al. 2010; Sica and Mantovani 2012). Considering these facts, we set out to assess the changes in the expression of macrophage activation markers in order to determine potential immunomodulative properties of ezetimibe, which is used in hypercholesterolemia treatment, in terms of the influence on atherosclerosis.

Numerous studies have demonstrated that stimulation of monocytic cells of various origins (human, animal, cell lines) with LPS results in an increase in IL-1β secretion (Netea et al. 2009; Okopień et al. 2005). In experiments on isolated human monocytes/macrophages stimulated with LPS in conditions similar to ours, the cells secreted comparable amounts of cytokine (Okopień et al. 2005). However, the influence of ezetimibe on IL-1β secretion in vitro is not well established. Until now, the drug has been observed to reduce the secretion of IL-1β in vivo (Alvarez-Sala et al. 2008; Undas et al. 2011). Despite obtaining information on the systemic inflammatory state in this way, it is not possible to precisely determine the source of the cytokine. In the experiments in which the patients were administered ezetimibe for 30 and 90 days, followed by isolation of monocytes, the secretion of the cytokine by the cells was reduced by 17 and 18 %, respectively. However, the observed differences were not statistically significant. Only the addition of simvastatin to ezetimibe was found to result in a significant reduction of IL-1β secretion (Krysiak and Okopień 2011). Nevertheless, in these experiments, ezetimibe was not used to stimulate the monocytes after isolation, which might have affected the final outcome. In recent in vitro experiments on isolated monocytes (Krysiak and Okopień 2011; Moutzouri et al. 2012), cells were not stimulated with ezetimibe thus interrupting their constant contact with the drug. The investigations in this study allowed for direct determination of the influence of ezetimibe on the IL-1β level in the inflammatory conditions in vitro and confirmed that the drug reduces its secretion.

As in the case of IL-1β, information on the influence of ezetimibe on the NO-iNOS-Arg1 balance is limited. Mostly, the impact of the drug on the endothelial NOS (eNOS) isoform has been reported. In the animal model, ezetimibe inhibited atherosclerosis progression and the effect was related with marked eNOS mRNA and protein expression upregulation (Nakagami et al. 2009). The data concerning iNOS expression was obtained in experiments on rats suffering from nonalcoholic fatty liver disease. Administering ezetimibe to the animals receiving a cholesterol-rich diet reduced liver iNOS expression (Ibrahim et al. 2010), which is in agreement with our results; however, the authors did not assess the expression of the enzyme, in particular types of cells, and they used whole-tissue homogenates.

Interestingly, in our experiments, ezetimibe did not influence the generated NO concentration in comparison to LPS stimulation. Simultaneously, at the 48-h timepoint, the expression of iNOS was more than twofold lower. This may suggest that LPS was initially predominant in the inflammatory reactions, thus resulting in increased iNOS expression and NO production. Next, during later timepoints of stimulation, ezetimibe restricted iNOS expression, but the derivatives of the generated NO still remained in the cell culture medium. As the concentration of NO produced in the co-stimulated cultures after 48 h was slightly (but not yet significantly) lower than in the LPS-stimulated cultures and the iNOS expression was markedly decreased, we conclude that this was probably the moment from which the activity of the drug overwhelmed the activity of LPS in this aspect. To confirm this, it will be necessary to perform a time-dependent study of iNOS expression and NO production.

As in our experiments, it was previously noted that LPS contributes to elevated Arg1 expression, which is an alternative macrophage activation marker (Rajaiah et al. 2013). It has been established by other authors that LPS is a classical macrophage activator. Despite this fact, they have also observed that, to some extent, LPS contributes to increased Arg1 expression. Furthermore, Arg1 expression in macrophages increases along with increasing LPS concentration (Sonoki et al. 1997). Concomitant iNOS and Arg1 expression upregulation is explained as a protection mechanism of the cells from iNOS overexpression and toxic NO influence. Arg1 is considered to be an enzyme that limits the NO concentration in the cell (Mielczarek-Puta et al. 2008). Interestingly, in our study, ezetimibe did not significantly increase the expression of Arg1 in the co-stimulated cells compared to the LPS stimulation. We consider this effect to be related to the reported lack of ezetimibe influence on the activity of the central pro-inflammatory regulator nuclear factor-κB (NF-κB) in human mononuclear cells, expression of which markedly increases after LPS treatment (Rudofsky et al. 2012). Apart from the pro-inflammatory features of NF-κB, its phosphorylation also contributes to elevated Arg1 expression (Mielczarek-Puta et al. 2008). This may explain the minor influence of ezetimibe on Arg1 expression compared to the LPS effect. However, the action of ezetimibe on NF-κB requires further investigation. Finally, it should be noted that for a significant increase in Arg1 mRNA expression, it was necessary to stimulate the cells with both LPS and ezetimibe, indicating that ezetimibe has an additional effect on the expression of this macrophage alternative activation marker.

The influence of LPS on the MR expression was insignificant (Ferrante et al. 2013; Rajaiah et al. 2013), which we also observed in our experiments. Importantly, elevated MR expression was noted in the alternatively activated macrophages localized in the atherosclerotic plaques (Wilson 2010). As yet, the influence of ezetimibe in vitro or in vivo on the expression of this marker has not been assessed. Other pharmacologically active substances such as eplerenone have demonstrated that induction of alternative macrophage activation is related to increased MR expression. Similarly to ezetimibe, the mineralosteroid receptor antagonist, elevated the expression of MR mRNA and protein in human monocyte-derived macrophages (Łabuzek et al. 2013). Moreover, administering eplerenone to Apo-E-knockout mice resulted in a restriction of the area with early atherosclerotic changes in the arch of the aorta, which was attributed to the change in the macrophage phenotype towards alternative activation (Raz-Pasteur et al. 2012). We observed that ezetimibe strongly increased MR expression in human monocyte-derived monocytes in vitro.

The quantitative and qualitative effects of macrophage stimulation by LPS observed in our study were similar to those noted in recent studies. This study is the first assessment of the influence of ezetimibe on the magnitude of macrophage activation marker expression. We confirmed that the drug presents anti-inflammatory properties, in particular resulting from a direct influence on macrophages. It can be assumed that ezetimibe may exert a local anti-inflammatory effect in growing atherosclerotic plaques, where the macrophages reside. We also presume that in the in vivo conditions—in which the atherosclerotic plaque is affected by constantly circulating fluids—the NO, once produced, would not accumulate locally. As further production of NO would be hampered due to decreased iNOS expression caused by ezetimibe, generation of reactive nitrogen species (RNS), which contribute to the modification of LDLs, would also be restricted. Therefore, the drug is probably able to restrict atherogenic conditions, stating an extra-lipid effect. Additionally, ezetimibe is likely to alter transmembrane trafficking due to reduction of MR expression. The drug may then not only effectively decrease the LDL level but may also exert a direct extra-lipid effect on the cells taking part in atherosclerotic plaque growth and stability modulation. Partial in vivo confirmation of the outcome of our investigations was observed in the experiments on rabbits. Administering ezetimibe to the animals resulted in the reduction of the atherosclerotic change areas in the femoral arteries. The drug also decreased the number of macrophages and the expression of monocyte chemoattractant protein-1 (MCP-1) in the atherosclerotic plaques. Additionally, ezetimibe was observed to reduce lipid-rich areas, preserving the collagen content (Gómez-Garre et al. 2009).

The analyzed markers are essential for the determination of macrophage activation pathways and also have an influence on atherosclerotic plaque stability. However, the biochemical mechanisms of macrophage phenotype alternation by ezetimibe still remain vague. Based on our current knowledge, macrophage phenotype modulation by ezetimibe might be due to its influence on the expression of Toll-like receptors (TLRs). The drug decreases TLR2 and TLR4 expression in the cell membranes of hypercholesterolemic patients. Signaling via both the receptors promotes inflammatory responses and progression of atherosclerosis. Furthermore, TLR2 and TLR4 expression is elevated in the monocytes of atherosclerotic patients (Moutzouri et al. 2012). Investigation of further activation markers such as tumor necrosis factor alpha, IL-6, IL-12, and reactive oxygen species for the classical activation and IL-10, tumor growth factor-β1, and prostaglandin-E2 for the alternative activation would be helpful to clarify the effect of ezetimibe and elucidate its potential mechanism.

## Conclusions

In summary, our study indicates that ezetimibe, used in vitro at a concentration that occurs in serum, is likely to induce the alternative activation of monocyte-derived macrophages isolated from patients with hypercholesterolemia, concomitantly suppressing the classical activation. Earlier studies additionally demonstrated that the drug increases the stability of atherosclerotic plaques in animals. Taken together, it is probable that the substance may stabilize the plaques in hypercholesterolemic patients. It is essential to carry out further clinical studies to assess the latter.
